# The Effects of Baclofen for the Treatment of Gastroesophageal Reflux Disease: A Meta-Analysis of Randomized Controlled Trials

**DOI:** 10.1155/2014/307805

**Published:** 2014-10-20

**Authors:** Shujie Li, Shengying Shi, Feng Chen, Jingming Lin

**Affiliations:** Department of Pharmacy, Zhujiang Hospital, Southern Medical University, No. 253 Industrial Road, Haizhu District, Guangzhou 510282, China

## Abstract

*Objectives*. Baclofen can relieve gastroesophageal reflux-related symptoms in healthy subjects and gastroesophageal reflux disease (GERD) patients by reducing the incidence of transient lower esophageal sphincter relaxation. This meta-analysis aimed to evaluate the efficacy and safety of baclofen for the treatment of GERD. *Methods*. We systematically searched randomized controlled trials published prior to November 2013 from PubMed, Medline, Embase, ScienceDirect, ClinicalTrials.gov, and the Cochrane Central Register of Randomized Controlled Trials. We performed a meta-analysis of all eligible trials. *Results*. Nine studies were identified with a total of 283 GERD patients and healthy subjects. Comparative analysis provided high quality data supporting the ability of baclofen to promote a short-term decrease in the number of reflux episodes per patient, the average length of reflux episodes, and the incidence of transient lower esophageal sphincter relaxation. No serious adverse events or death events were reported, and there were no significant differences in the overall adverse events between baclofen and placebo. All reported side effects of baclofen were of mild-to-moderate intensity, and the drug was well tolerated. *Conclusion*. Abundant evidence suggests that baclofen may be a useful approach for the treatment of GERD patients; however, a larger well-designed research study would further confirm this recommendation.

## 1. Introduction

Gastroesophageal reflux disease (GERD), which is defined as a disorder caused by the reflux of gastric contents into the esophagus, has long been a prominent concern worldwide. Gastric reflux can evoke aggravating symptoms, such as heartburn and regurgitation, and additional complications, such as erosive esophagitis, can also occur [1–3]. The disease can be classified into three subtypes: nonerosive reflux disease, hypersensitive esophagus, and functional heartburn [4]. Endoscopic or microscopic evidence of damage to the esophageal mucosa can be observed for GERD patients, though the body undergoes initial attempts to protect itself by tightening the gastroesophageal junction, a muscular complex consisting of the lower esophageal sphincter, the rural diaphragm, and the gastric sling [5, 6]. Recent evidence suggests that transient lower esophageal sphincter relaxation (TLESR) might be the primary cause of reflux episodes in patients with GERD [7, 8].

Proton pump inhibitors [9] and histamine type 2 receptor antagonists [10] are first-line treatment for patients with GERD. Both methods depend primarily on the inhibition of acid secretion. Despite their high performance in symptom resolution and esophageal mucosal healing, clinical failure has become a common dilemma for patients with GERD [11]. The primary reason for the clinical failure may be the inability of these agents to control TLESR.

As an alternate approach to the treatment of GERD, baclofen, a GABA_B_ agonist, reduces the frequency of reflux events and inhibits TLESR [8]. Numerous randomized controlled trials (RCTs) over the last decade have pointed to the therapeutic efficacy of baclofen for GERD. However, most of these studies are of limited size, and, therefore, the role of baclofen in the treatment of GERD remains unsupported. In this study, a meta-analysis of relevant RCTs [12–20] was performed to support the clinical efficacy and safety of baclofen for the treatment of GERD.

## 2. Methods

### 2.1. Data Sources

We performed an independent review of Medline, PubMed, ScienceDirect, and Embase databases to identify RCTs from January 1978 to November 2013 using “baclofen” and “GERD” as search key words. The search was limited to human studies and RCTs published in English. We also manually searched abstracts and full-text articles containing the same search terms from ClinicalTrials.gov and the Cochrane Central Register of Controlled Trials to identify potentially relevant RCTs that were published before November 2013. An independent search of Google Scholar was also conducted to ensure that no clinical trials had been left out. To find additional articles relevant to the content of our meta-analysis, references from potentially relevant articles were also individually researched [21, 22]. Studies were selected and systemically reviewed according to the Preferred Reporting Items for Systematic Reviews and Meta-Analyses (PRISMA) statement [23].

### 2.2. Study Selection/Inclusion Criteria

We selected studies for this meta-analysis according to the following criteria: (1) studies were randomized double-blind trials that compare baclofen and placebo for GERD; (2) studies determined the efficacy and safety of baclofen for the treatment of GERD; (3) studies reported specific data regarding symptomatic relief and adverse events. Abstracts of scientific conferences were excluded in the meta-analysis, as well as trials that focused on pharmacokinetic or pharmacodynamic variables. We included both single dose and multiple dose/crossover studies. We also included studies in which baclofen was given either alone or as an addition to proton pump inhibitors.

### 2.3. Data Extraction

Two investigators (SJ. Li and SY. Shi) independently screened data from trials according to the inclusion criteria. We extracted data from the studies, including the type of study, the patients enrolled, the per protocol (PP) population, the mean age, the dosing regimen, the rate of gastroesophageal reflux episodes (GER) in the PP population, the acid reflux time, the drug-related adverse events (AEs), the serious AEs, the serious drug-related AEs, and mortality. Any disagreements in extracted data between the two reviewers were resolved by discussion among all of the authors.

### 2.4. Quality Assessment

We assessed the methodological quality of RCTs using the Jadad criteria. Three items were considered for the Jadad scale: (1) whether the study was described as randomized; (2) whether the study was described as doubled-blind; (3) whether a description of drop-outs and withdrawals was provided. One point was awarded for each of these items that had a positive answer. One point was awarded to the study if the randomization procedure was considered appropriate, and one point was deducted if the randomization procedure was considered inadequate. Similarly, one point was awarded to the study if the blinding was considered appropriate, and one point was deducted if the blinding was considered inadequate. Five points were the maximum score that could be assigned to a trial, and scores higher than 2 were deemed to be indicative of adequate methodological quality [24–26].

### 2.5. Statistical Analysis

This meta-analysis was performed using Review Manager 5.1, which was provided by Cochrane.org. Meta-analysis methods were used to combine data obtained from separate trials. Results were pooled for sufficiently similar outcomes and homogeneous data (which were determined by the degree of statistical heterogeneity). The *χ* ^2^ test was used to evaluate statistical heterogeneity between trials, with significance regarded as a* P* value = 0.10. For dichotomous data, the Mantel-Haenszel fixed-effects model was used to calculate the pooled odds ratio and 95% confidence intervals (CI) when there was no statistically significant heterogeneity between the included trials (heterogeneity *P* > 0.1). When heterogeneity of *P* < 0.1 and *I* ^2^ > 50% was found among the included studies, a random-effect's model was chosen. If there was no heterogeneity detected by this method, the* I* ^2^ test was used. If the heterogeneity of *I* ^2^ > 70% was evident, the inferior quality study was excluded from the meta-analysis.

## 3. Results

### 3.1. Study Selection Process

**Figure 1:**
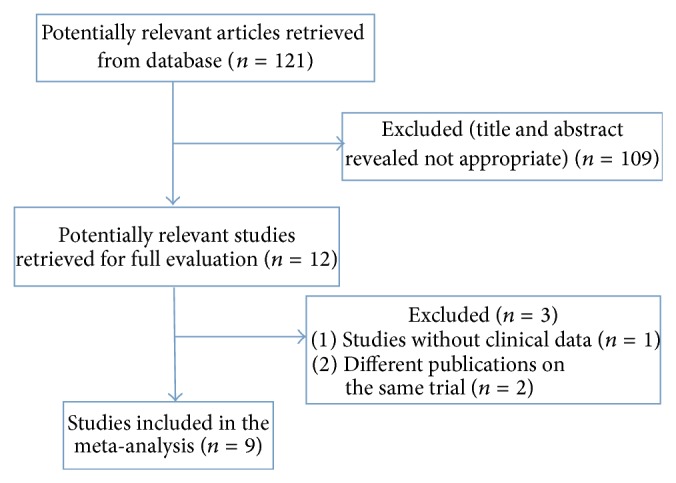
Flow diagram showing the procedure for the systematic review of studies for meta-analysis.

We identified 121 articles through database searching after application of our criteria. Two records were excluded because of duplication. Of the remaining 119 articles, nine [12–20] RCTs were selected for meta-analysis based on the inclusion criteria. The search process is summarized in Figure 1. The same searching results were reached by the two independent reviewers.

### 3.2. Study Characteristics

All nine trials were double-blinded RCTs, and four of them were crossover studies. The trials selected for this study were conducted in primary and secondary care settings in different countries and represented a total of 283 GERD patients and healthy subjects. One trial assessed baclofen as an “add on” therapy to proton pump inhibitors [12], but the other nine trials assessed baclofen as an individual therapy. One trial adopted a prodrug of the active R-isomer of baclofen. Because the mechanism of action is identical, that study was also included [13]. The subjects in the studies had nonerosive reflux disease, hypersensitive esophagus, or functional heartburn, all of which characterize GERD. Most patients were adults in their 40s (except for 30 children in their 10s). The treatment duration varied from 12 h to 4 weeks according to the designs for each trial. The details of the nine RCTs, including study design, parameters evaluated, number of patients, mean age, study duration, and dosing regimens, are summarized in Table 1.

Quality assessment of the nine RCTs is summarized in Table 2. All nine RCTs were assigned a Jadad score >2. Of those, two trials were assigned a Jadad score of 5, three trials were assigned a Jadad score of 4, and the remaining ones were assigned a Jadad score of 3.

### 3.3. Reduction in the Incidence of GER

**Figure 2:**
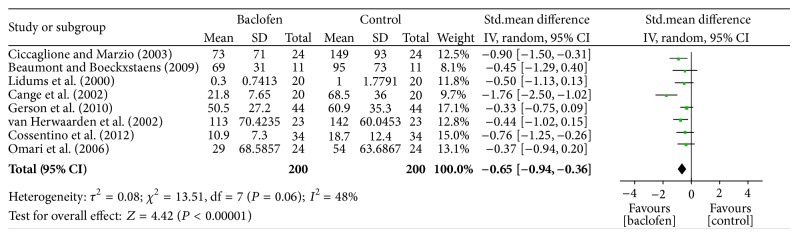
Meta-analysis of the incidence of GER in the PP population given either baclofen or placebo for the treatment of GERD.

Data regarding the effect of baclofen on the incidence of GER in the PP group were provided by eight of the nine RCTs. Data for nonerosive reflux disease, hypersensitive esophagus, and functional heartburn were measured by pH metry, manometry, and symptom assessment, respectively. We observed a statistically significant difference in the reduction in GER incidence between baclofen-treated and placebo-treated subjects (standardized mean difference [SMD]: −0.65; 95% CI: −0.94, −0.36; *P* = 0.00001); moreover, the statistical heterogeneity was insignificant (*I* ^2^ = 48%; *P* = 0.06) (Figure 2). These results provide confirmation that baclofen is effective in reducing the incidence of GER.

### 3.4. The Acid Reflux Time in the PP Population Who Were Given Either Baclofen Or Placebo for the Treatment of GERD

**Figure 3:**
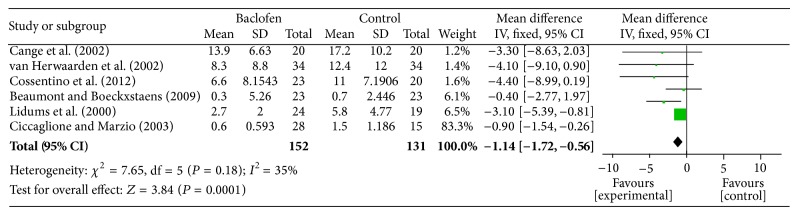
Meta-analysis of the acid reflux time in the PP population given either baclofen or placebo for the treatment of GERD.

Data regarding the effect of baclofen on the acid reflux time in the PP group were provided by six of the nine RCTs [13–16, 18, 20]. We identified a statistically significant difference between baclofen and placebo (SMD: −1.14; 95% CI: −1.72, −0.56; *P* = 0.00001), and the statistical heterogeneity was insignificant (*I* ^2^ = 35%; *P* = 0.18) (Figure 3). These results provide confirmation that baclofen decreases the acid reflux time for GERD patients.

### 3.5. The Rate of TLESR in the PP Population Who Were Given Either Baclofen Or Placebo for the Treatment of GERD

**Figure 4:**
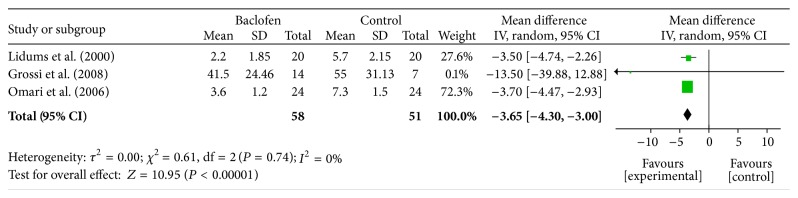
Meta-analysis of the incidence of TLESR in the PP population given either baclofen or placebo for the treatment of GERD.

Data regarding the effect of baclofen on the incidence of TLESR in the PP group were provided by three of the nine RCTs [17–19]. A statistically significant difference was detected between baclofen- and placebo-treated subjects for decreasing the rate of TLESR (SMD: −3.65; 95% CI: −4.30, −3.00; *P* < 0.00001), and the statistical heterogeneity was insignificant (*I* ^2^ = 0%; *P* = 0.73) (Figure 4). These results verify that baclofen decreases the incidence of TLESR.

### 3.6. Side Effects in the PP Population Who Were Given Either Baclofen Or Placebo for the Treatment of GERD

**Figure 5:**
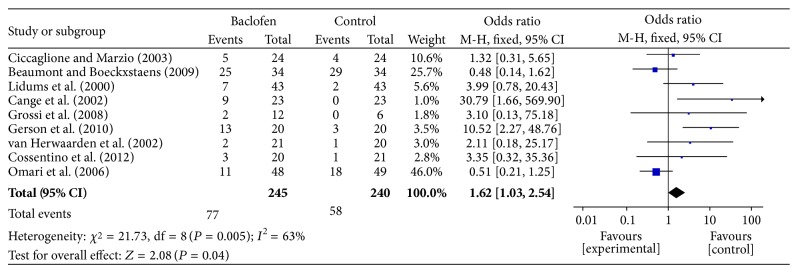
Meta-analysis of the overall adverse events in the PP population given either baclofen or placebo for the treatment of GERD.

Data for the overall adverse events of baclofen and placebo in the PP group were provided by all nine RCTs. There was no statistically significant difference in the frequency of overall AEs between subjects given baclofen and those given placebo (OR = 1.62; 95% CI: 1.03–2.54; *P* = 0.04), and the statistical heterogeneity was high (*I* ^2^ = 63%, *P* = 0.005) (Figure 5). Associated mortality was not observed in any of the nine RCTs included in this analysis. All side effects reported in the studies were of mild-to-moderate intensity. Mental/neurological symptoms (dizziness, tiredness, sleepiness, and accommodation disorder) were most commonly reported as a side effect. Other reported side effects were abdominal complaints (discomfort, nausea, diarrhea, and flatulence) and pain (headache, muscular). These results suggest that baclofen does not significantly increase the number of AEs.

## 4. Discussion

### 4.1. Summary of Main Results

This meta-analysis provides highly statistical confirmation that baclofen is effective for the relief of GERD-related symptoms. Baclofen treatment was associated with a significant reduction in the number of GER episodes, the acid reflux time, and the incidence of TLESR. Our meta-analysis also demonstrated that there is no statistically significant difference in the occurrence of the overall adverse events between baclofen- and placebo-treated subjects and that the drug was well tolerated.

### 4.2. Applicability of the Evidence

All trials included in the meta-analysis provided categorical information about the types of GERD-related symptoms (the incidence of TLESR, GER, gastric emptying, pharyngeal swallowing, and lower esophageal sphincter pressure and the acid reflux time). The mean incidence of TLESR and GER and the acid reflux time were decreased among studies by different treatment. When meta-analysis was carried out to verify the efficacy of baclofen on GERD-related symptoms, the mean differences between baclofen and placebo became smaller, but greater statistical significance was achieved (SMD: −0.65; 95% CI: −0.94, −0.36; *P* = 0.00001), (SMD: −1.14; 95% CI: −1.72, −0.56; *P* = 0.00001), and (SMD: −3.65; 95% CI: −4.30, −3.00; *P* < 0.00001). Therefore, the meta-analysis provides more reliable data to support the positive effects of baclofen.

### 4.3. Agreements and Disagreements with Other Systematic Reviews

A thorough literature search located one other review of baclofen for the treatment of GERD [26], which was a systematic review, rather than a meta-analysis, and included only five studies on baclofen for the treatment of GERD with only adult patients. This review concluded that baclofen produces statistically significant reduction in various objective measures of reflux but is not associated with symptomatic improvement and produces mild adverse effects.

Given that nine RCTs were included in this meta-analysis and that the evidence to support the effects of baclofen was determined in comparison to placebo, rather than active controls, we conclude that baclofen might be effective in the short term. Unfortunately, with regard to long-term efficacy, our meta-analysis does not allow for conclusions.

### 4.4. Strengths and Weaknesses

Many studies have shown that baclofen can reduce GER episodes [27, 28] and decrease the acid reflux time and the incidence of TLESR [29, 30] in normal individuals and patients with GERD. The mechanism of action of baclofen in reducing reflux involves the inhibition of TLESR, which is different from proton pump inhibitors that reduce reflux by inhibiting acid secretion [31–33]. The relaxation of the lower esophageal sphincter is one of the primary causes of reflux events [34–36]. The effect of baclofen in reducing reflux can last almost 24 h. Therefore, baclofen has already been suggested as a primary or adjunct treatment for GERD [37, 38], especially for disease that has failed to respond to proton pump inhibitors and histamine type 2 receptor antagonists. This meta-analysis is the first to pool clinical data from numerous double-blinded RCTs on baclofen for the treatment of GERD and to investigate the efficacy and safety of baclofen. It will provide useful reference data for clinical practice.

There are several weaknesses of our meta-analysis that should be taken into account when we evaluate the results. First, the study's primary limitation is the paucity of eligible trials, which prohibited further subgroup analyses. Second, most of the research included in this study had poor methodological quality and/or small sample size. Finally, additional studies comparing baclofen to other active therapies and with significant sample size are urgently needed.

## 5. Conclusions

Although there are some limitations of this meta-analysis, treatment with baclofen was demonstrated to significantly result in the improvement of GERD-related symptoms. Moreover, compared with placebo, baclofen did not increase the number of severe adverse events in patients with GERD. Additional well-designed RCTs are needed to confirm these conclusions.

## Figures and Tables

**Table 1:** Characteristics of the baclofen studies included in this meta-analysis.

Source	Study design	Parameters evaluated	Number of patients	Mean age	Study duration	Dosing regimen
Beaumont and Boeckxstaens (2009) [12]	DB, RCT crossover	Incidence of GER, acid reflux time	27	54.0	12 d	T.i.d 20 mg
Cange et al. (2002) [13]	DB, RCT crossover	Incidence of acid GER, acid reflux time	20	41.2	4 w	Q.d 40 mg
Ciccaglione and Marzio (2003) [14]	DB, RCT	Incidence of GER, acid reflux time	43	41.0	4 w	Q.i.d 10 mg
	DB, RCT	Incidence of GER, acid reflux time	43	49.0	2 w	T.i.d
						10, 10, 10 mg (D1–3)
Cossentino et al. (2012) [15]						20, 10, 10 mg (D4)
						20, 20, 10 mg (D5)
						20, 20, 20 mg (D6–14)
Gerson et al. (2010) [16]	DB, RCT crossover	Incidence of GER, heartburn	50	41.0	4 w	Q.d, 4 different doses
						10, 20, 40, 60 mg
Grossi et al. (2008) [17]	DB, RCT	Incidence of TLESR	23	43.0	2 d	Q.i.d 10 mg
Lidums et al. (2000) [18]	DB, RCT	Incidence of GER, incidence of TLESR, acid reflux time, and rate of swallowing	20	24.0	1 w	Q.2d 40 mg
Omari et al. (2006) [19]	DB, RCT^1^	Incidence of TLESR, incidence of GER, gastric emptying, pharyngeal swallow, LES pressure, and acid reflux time	30	10.0	12 h	A single dose 0.5 mg/kg
van Herwaarden et al. (2002) [20]	DB, RCT crossover	Incidence of TLESR, incidence of GER, and acid reflux time	37	42.3	4 days	Q.d 40 mg

DB: double-blinded; GER: gastroesophageal reflux; RCT: randomized controlled trial; Q.d: once per day; T.i.d: 3 times per day; Q.i.d: 4 times per day; Q.2d: once per 2 days.

**Table 2:** Quality assessment of RCTs in this study.

Source	Randomization	Blinding	Withdrawals and dropouts	Jadad score
Beaumont and Boeckxstaens (2009) [12]	1	1	1	3
Cange et al. (2002) [13]	1	1	1	3
Ciccaglione and Marzio (2003) [14]	1	2	1	4
Cossentino et al. (2012) [15]	2	2	1	5
Gerson et al. (2010) [16]	2	2	1	5
Grossi et al. (2008) [17]	1	2	1	4
Lidums et al. (2000) [18]	1	1	1	3
Omari et al. (2006) [19]	1	2	1	4
van Herwaarden et al. (2002) [20]	1	1	1	3
